# Burden of cerebral small vessel disease and changes of diastolic blood pressure affect clinical outcome after acute ischemic stroke

**DOI:** 10.1038/s41598-023-49502-6

**Published:** 2023-12-12

**Authors:** Sarah Gunkel, Andreas Schötzau, Felix Fluri

**Affiliations:** 1 Department of Neurology, University Hospital Würzburg, Josef-Schneider Strasse 11, 97080 Würzburg, Germany; 2 Eudox Statistics, Basel, Switzerland; 3 Department of Biomedicine, University of Basel, Basel, Switzerland

**Keywords:** Neurological disorders, Cerebrovascular disorders, Stroke, White matter disease

## Abstract

Elevated and low blood pressure (BP) may lead to poor functional outcome after ischemic stroke, which is conflicting. Hence, there must be another factor—such as cerebral small vessel disease (cSVD) -interacting with BP and thus, affecting outcome. Here, we investigate the relationship between BP and cSVD regarding outcome after stroke. Data of 423/503 stroke patients were prospectively analyzed. Diastolic (DBP) and systolic BP (SBP) were collected on hospital admission (BP_ad_) and over the first 72 h (BP_72h_). cSVD-burden was determined on MR-scans. Good functional outcome was defined as a modified Rankin Scale score ≤ 2 at hospital discharge and 12 months thereafter. cSVD was a predictor of poor outcome (OR 2.8; *p* < 0.001). SBP_ad_, DBP_ad_ and SBP_72h_ were not significantly associated with outcome at any time. A significant relationship was found between DBP_72h_, (*p* < 0.01), cSVD (*p* = 0.013) and outcome at discharge. At 12 months, we found a relationship between outcome and DBP_72h_ (*p* = 0.018) and a statistical tendency regarding cSVD (*p* = 0.08). Changes in DBP_72h_ were significantly related with outcome. There was a U-shaped relationship between DBP_72h_ and outcome at discharge. Our results suggest an individualized stroke care by either lowering or elevating DBP depending on cSVD-burden in order to influence functional outcome.

## Introduction

Increased blood pressure (BP) is found in around 80% of all patients with acute ischemic stroke (AIS) on hospital admission as well as in the acute phase of AIS^1^. However, the impact of BP in the acute phase of AIS on functional outcome remains an matter of debate^2–4^. So far, there is some evidence that raised BP early after AIS is associated with dependency, clinical deterioration and subsequent death^5^. In contrast, some studies revealed that both low and high BP entails poor prognosis after AIS^4,6^. Further studies found no effect of BP in the acute phase of AIS on functional outcome^7,8^. Of note, some other studies even suggested that an increase of BP in the acute phase of AIS may be a protective response to reduced cerebral perfusion and thus, may enhance decreasing blood flow to the infarcted zone and surrounding penumbra^9–11^. Hence, these different and partially contradictory findings suggest that additional factors such as cerebral small vessel disease (cSVD) may interfere prognosis and clinical outcome after AIS. The term cSVD refers to the dysfunction of cerebral microvessel endothelium affecting cell–cell interactions and finally resulting in brain damage^12–14^. Clinically, cSVD may lead to stroke, cognitive dysfunction and worsening of gait as well as balance^15^. On brain MRI, cSVD presents with lacunes, white matter hyperintensities (WMH), cerebral microbleeds (CMB) and perivascular spaces^12,15,16^. In particular, WMH are the most common cSVD lesions that are observed on brain MR scans of most people aged over 70 years^17^. CMB also increase with age although they occur less frequent compared to WMH and are unusual in the absence of WMH^13^. In a recently published study on stroke patients undergoing endovascular treatment, no signs of cSVD was observed in 61%, mild to moderate and severe degree of cSVD was seen in 20.0% and 19.7%, respectively^18^. Hence the question arises whether patients with AIS and no cSVD profit e.g., from a lower BP than stroke patients with a moderate or severe degree of cSVD. Of note, autoregulation of cerebral blood flow is hampered in the acute phase of AIS making cerebral perfusion directly dependent on BP^19–21^. Additionally, patients with severe cSVD have an even more reduced cerebral blood flow and impaired cerebral autoregulation^22,23^.

With these considerations in mind, we aimed to investigate whether functional outcome in the short term (at discharge) and in the long term (12 months after cerebrovascular event) differs between stroke patients with a high and low burden of cSVD depending on the level of acute-phase systolic (SBP) and/or diastolic blood pressure (DBP). Additionally, we examined whether there is an interaction between BP in the acute phase and severity of cSVD regarding functional impairment, recurrent stroke/TIA and death 12 month after index event.

## Methods

### Study design and setting

We conducted a prospective single-center cohort study at the University Clinic Würzburg (Stroke Unit, Department of Neurology). From October 2016 to October 2017, all consecutive patients with an ischemic stroke or transient ischemic attack (TIA) admitted to the emergency room within 24 h after onset of symptoms were included in the study. Written informed consent from the patient or patients’ next of kin was obtained before enrolment. The study was approved by the local ethics committee of Würzburg (AZ 223/16). The study protocol adheres to the established standards for the reporting of observational studies^24^. Anonymous data will be shared by request.

### Data collection

During hospitalization, data on demographic characteristics (age, sex), vascular risk factors (hypertension, diabetes mellitus, dyslipidemia, coronary heart disease, myocardial infarction, atrial fibrillation, previous stroke or TIA, peripheral artery occlusive disease and current cigarette smoking), comorbidities (liver and kidney dysfunction), previous medication (antihypertensives, antiplatelets, anticoagulants, statins), previous degree of disability (according to modified Rankin scale (mRS)), recanalization therapy (intravenous thrombolysis; endovascular treatment), discharge medication (antihypertensives, antiplatelets, anticoagulants, statins) and stroke/TIA characteristics were collected prospectively in a database (Table 1). Stroke characteristics included (1) stroke severity on admission and at discharge assessed by the National Institutes of Health Stroke Scale (NIHSS) score^25^ and (2) stroke subtype classified according to the criteria of Trial of Org 10,172 in Acute Stroke Treatment (TOAST)^26^.

**Table 1:** Baseline characteristics of stroke patients regarding outcome at discharge. mRS, modified Rankin Scale, SBP, systolic blood pressure; DBP, diastolic blood pressure, CMB, cerebral microbleeds, WML, white matter lesions; cSVD cerebral small vessel disease, NIHSS, National Institutes of Health Stroke Scale; TIA, transient ischemic attack; SD, standard deviation, p-values calculated with chi2-test.

	All patients	Good outcome (mRS 0–2)	Poor outcome (mRS 3–6)	*p*-value
n	423	311	112	
Demographics				
Age, median (Q1-Q3), y, n = 423	73 (61–79)	70 (59–79)	78 (65.8–83)	< 0.001^1^
Female, n = 423 (%)	184 (43.5%)	127 (40.8%)	57 (50.9%)	0.084
Vascular risk factors, n (%)				
Hypertension, n = 422	359 (85.1)	256 (82.3)	103 (92.8)	0.012
Newly diagnosed hypertension, n = 422	31 (7.35)	22 (7.07)	9 (8.11)	0.883
Atrial fibrillation, n = 422	114 (27.0)	72 (23.2)	42 (37.8)	0.004
Current Smoking, n = 422	74 (17.5)	58 (18.6)	16 (14.4)	0.389
Hypercholesterolemia, n = 422	238 (56.4)	180 (57.9)	58 (52.3)	0.360
Diabetes mellitus, n = 422	97 (23.0)	61 (19.6)	36 (32.4)	0.009
Coronary heart disease, n = 422	56 (13.3)	32 (10.3)	24 (21.6)	0.004
Myocardial Infarction, n = 422	23 (5.45)	13 (4.18)	10 (9.01)	0.093
Prior stroke or TIA, n = 422	103 (24.4)	69 (22.2)	34 (30.6)	0.099
Positive Family history, n = 422	101 (23.9)	76 (24.4)	25 (22.5)	0.782
Peripheral artery occlusive disease, n = 422	23 (5.45)	11 (3.54)	12 (10.8)	0.008
Kidney dysfunction, n = 422	135 (32.0)	86 (27.7)	49 (44.1)	0.002
Liver dysfunction, n = 422	7 (1.66)	2 (0.64)	5 (4.5)	0.015
Clinical data				
NIHSS at entry, median (Q1-Q3), n = 423	3 (1–6)	2 (0–4)	7 (4–12.2)	< 0.001^1^
NIHSS at discharge, median (Q1-Q3), n = 423	1 (0–3)	0 (0–1)	4 (2–7)	< 0.001^1^
mRS at entry, mean ± SD, n = 423	2.37 ± 1.58	1.77 ± 1.35	4.0 ± 0.76	< 0.001
mRS at discharge, mean ± SD, n = 423	1.44 ± 1.43	0.71 ± 0.81	3.45 ± 0.6	< 0.001
Recanalization therapy, n (%)				
Intravenous thrombolysis, n = 423	91 (21.5)	54 (17.4)	37 (33.0)	< 0.001
Endovascular treatment, n = 423	37 (8.75)	15 (4.82)	22 (19.6)	< 0.001
MRI Findings, n (%)				
Lacunes, n = 412	172 (41.7)	119 (39.0)	53 (49.5)	0.074
WML (Fazekas score 2–3) , n = 414	228 (55.1)	154 (50.5)	74 (67.9)	0.003
CMB, n = 419	113 (27.0)	76 (24.6)	37 (33.6)	0.087
cSVD (CMB a./o.WML) , n = 419	259 (61.8)	173 (56.0)	86 (78.2)	< 0.001
Stroke etiology, n = 423, n (%)				
Large artery disease	79 (18.7)	59 (19.0)	20 (17.9)	0.115
Cardioembolism	160 (37.8)	107 (34.4)	53 (47.3)	0.221
Small vessel occlusion	30 (7.09)	23 (7.40)	7 (6.25)	0.849
Other determined	9 (2.13)	6 (1.93)	3 (2.68)	0.609
Undetermined	145 (34.3)	116 (37.3)	29 (25.9)	0.364

^1^Calculated with Mann–Whitney-U-test.

All patients underwent brain MRI within 72 h after admission to identify acute ischemic stroke or TIA. WMH were categorized visually on fluid-attenuated inversion recovery (FLAIR) scans using the Fazekas scale and rated as mild (Fazekas 0–1) or severe (Fazekas 2–3)^27^. CMB were defined as small, circular or rounded, hypointense lesions within brain parenchyma ranging from 2 to 10 mm in size^28^. Assessment of CMB was performed on T2*-weighted gradient-recalled echo (T2*-GRE) scans or on susceptibility-weighted images (SWI). Lacunes were defined as CSF-filled cavities with a diameter of at least 3 mm^29^. cSVD burden was visually rated on MR scans and divided in absent/mild cSVD (Fazekas 0–1, no CMB) and moderate/severe cSVD (Fazekas 2–3 and/or ≥ 1 CMB).

Blood pressure (BP) was measured on the non-paralytic arm in the supine position by doctors or trained nurses using a noninvasive BP monitoring device according to our in-hospital guidelines for acute stroke management. BP values obtained on patients’ admission and during their stay in the stroke unit were entered in the electronic health record system. From this health record system, we retrieved the very first SBP and DBP value of each patient at the time of admission (i.e., BP at entry). SBP and DBP values measured every 8 h during the first three days after hospital admission were also extracted from the electronic health record system. To obtain the mean SBP and DBP value for each of these three days, the three SBP and DBP values of day 1, 2 and 3 respectively, were averaged. Finally, the mean values of each day were averaged resulting in the mean SBP and DBP value over the first 72 h. In addition, patients with known and treated hypertension continued antihypertensive treatment, unless BP was below 120/70 mmHg (intern guidelines). When SBP at the stroke unit was > 160 mmHg, antihypertensive treatment was adjusted accordingly and BP was measured every 15 min., until SBP was below 160 mmHg. Patients without antihypertensive treatment until admission received angiotensin-converting enzyme inhibitor as the drug of first choice when SBP was > 160 mmHg. The number of patients with newly diagnosed arterial hypertension on hospital was 7.35%. Change in SBP or DBP over the time was calculated as SBP or DBP at time point—SBP or DBP at baseline (i.e. at hospital admission)^21^.

In order to evaluate functional outcome of TIA and stroke patients, the mRS score was assessed prospectively on discharge (outcome_disc_) and at 12 months after cerebrovascular event (outcome_12mo_) through a structured follow-up telephone interview by a trained stroke fellow (S.G.). During the interview, current medication and BP were collected. The mRS score on discharge as well as at 12 months was dichotomized into good (mRS score 0–2) and poor outcome (mRS score of 3–6)^30^.

### Statistical analysis

The baseline data of all patients are demonstrated as mean ± SD or median (first quartile, Q1; third quartile, Q3) for continuous variables or frequency (percentage) for categorical variables. First, study parameters were compared in a descriptive manner regarding good and poor functional outcome at discharge and at 12 months after stroke. Metric or ordinal variables have been compared with unpaired t-test, or Mann–Whitney-U-test as appropriate. For dichotomous traits like hypertension or diabetes mellitus, Chi-square-test was used. Second, multivariable logistic regression was used to estimate the influence of the different study variables in respect to functional outcome at discharge and at 12 months thereafter. Multivariable logistic regression was adjusted for age and sex as well as for each selected parameter (i.e., cSVD, mean DBP over 72 h). DBP and age were modelled in a non-linear way using a 3-knot restricted cubic spline, as described in detail elsewhere^31^. Results of the regression analysis were depicted as odds ratios (OR) and 95% confidence interval (95%CI). Possible interactions between selected variables were calculated using Likelihood-ratio Chi-square-tests. *P* < 0.05 was regarded statistically significant. Statistical analyses were performed using R version 4.0.4 (R: a Language and Environment for Statistical Computing; Vienna, Austria, 2021-03-15).

## Results

### Study population and baseline characteristics

**Figure 1:**
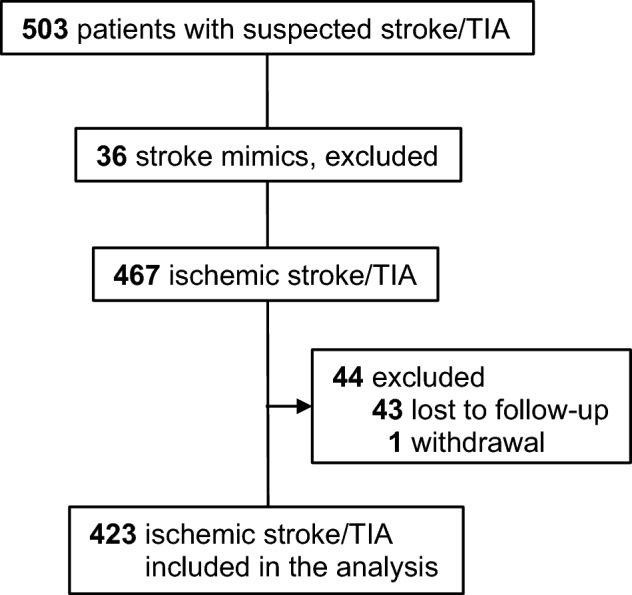
Flowchart of study participants.

A total of 503 patients with either ischemic stroke or TIA were screened for this study. Out of them, full data set was available for 423 patients at discharge (stroke-patients 76% (n = 322); TIA-patients 24% (n = 101)), and 369 patients completed follow-up 12 months after event (stroke-patients 75% (n = 276); TIA-patients 25% (n = 93)) (Fig. 1). The baseline characteristics for the final cohort are summarized in Tables 1 and 2.

**Table 2:** Baseline characteristics of stroke patients regarding outcome at 12 months. mRS, modified Rankin Scale, SBP, systolic blood pressure; DBP, diastolic blood pressure, CMB, cerebral microbleeds, WML, white matter lesions; cSVD, cerebral small vessel disease, NIHSS, National Institutes of Health Stroke Scale; TIA, transient ischemic attack; SD, standard deviation, p-values calculated with chi2-test.

	All patients	Good outcome (mRS 0–2)	Poor outcome (mRS 3–6)	*p*-value
n	369	261	108	
Demographics				
Age, median (Q1-Q3), n = 369, y	72 (60–79)	68 (58–77)	78 (69–83)	< 0.001^1^
Female, n = 369, n (%)	157 (42.5%)	104 (39.8%)	53 (49.1%)	0.106
Vascular risk factors, n (%)				
Hypertension, n = 369	310 (84.0)	214 (82.0)	96 (88.9)	0.137
Newly diagnosed hypertension, n = 369	25 (6.78)	20 (7.66)	5 (4.63)	0.408
Atrial fibrillation, n = 369	97 (26.3)	50 (19.2)	47 (43.5)	< 0.001
Current Smoking, n = 369	66 (17.9)	53 (20.3)	13 (12.0)	0.082
Hypercholesterolemia, n = 369	211 (57.2)	154 (59.0)	57 (52.8)	0.325
Diabetes mellitus, n = 369	86 (23.3)	51 (19.5)	35 (32.4)	0.012
Coronary heart disease, n = 369	50 (13.6)	28 (10.7)	22 (20.4)	0.022
Myocardial Infarction, n = 369	20 (5.42)	10 (3.83)	10 (9.26)	0.065
Prior stroke or TIA, n = 369	93 (25.2)	55 (21.1)	38 (35.2)	0.007
Positive Family history, n = 369	93 (25.2)	69 (26.4)	24 (22.2)	0.474
Peripheral artery occlusive disease, n = 369	23 (6.23)	7 (2.68)	16 (14.8)	< 0.001
Kidney dysfunction, n = 369	115 (31.2)	76 (29.1)	39 (36.1)	0.232
Liver dysfunction, n = 369	5 (1.36)	2 (0.77)	3 (2.78)	0.152
Clinical data				
NIHSS at entry, median (Q1-Q3) , n = 369	2 (1–5)	2 (0–4)	5 (2–9)	< 0.001^1^
NIHSS at discharge, median (Q1-Q3), n = 369	1 (0–3)	0 (0–2)	2 (0–6)	< 0.001^1^
mRS at entry, mean ± SD, n = 369	2.31 ± 1.58	1.87 ± 1.45	3.37 ± 1.38	< 0.001
mRS at discharge, mean ± SD, n = 369	1.38 ± 1.41	0.87 ± 1.06	2.61 ± 1.39	< 0.001
Recanalization therapy, n (%)				
Intravenous thrombolysis, n = 369	74 (20.1)	49 (18.8)	25 (23.1)	0.417
Endovascular treatment, n = 369	29 (7.86)	16 (6.13)	13 (12.0)	0.088
MRI Findings, n (%)				
Lacunes, n = 361	153 (42.4)	97 (37.7)	56 (53.8)	< 0.001
WML (Fazekas score 2–3), n = 368	200 (54.9)	126 (48.8)	74 (69.8)	0.003
CMB, n = 368	103 (28.0)	68 (26.1)	35 (32.7)	0.244
cSVD (CMB a./o.WML) , n = 368	229 (62.2)	145 (55.6)	84 (78.5)	< 0.001
Stroke etiology, n (%), n = 369				
Large artery disease	70 (19.0)	49 (18.8)	21 (19.4)	0.037
Cardioembolism	137 (37.1)	86 (33.0)	51 (47.2)	0.307
Small vessel occlusion	29 (7.86)	22 (8.43)	7 (6.48)	0.574
Other determined	7 (1.90)	4 (1.53)	3 (2.78)	0.508
undetermined	126 (34.1)	100 (38.3)	26 (24.1)	0.3149
Death within 1 year, n (%), n = 368	28 (7.61)	0	28 (25.9%)	< 0.001
Recurrent stroke within 1 year, n (%), n = 368	42 (11.4)	25 (9.58)	17 (15.9)	0.122

^1^Calculated with Mann–Whitney-U-test.

### Early functional outcomes at discharge

At hospital discharge, 311 (73.5%) patients had a good clinical outcome and 112 (26.5%) a poor outcome (Table 1). WMH (Fazekas score 2–3) occurred significantly more often in patients with a poor outcome (74 (67.9%) vs 154 (50.5%), *p* < 0.001), whereas CMB did not (37 (33.6% vs 76 (24.6%), *p* = 0.087). Previous lacunes were more common in patients with a poor outcome than in those with a good outcome at hospital discharge, however there was no significant difference between these two groups (119 (39%) vs 53 (49.5%), *p* = 0.07). When comparing the burden of cSVD among these two groups (i.e., WMH and/or CMB), patients with a poor outcome were significantly more often affected by cSVD (86 (78.2%) vs 173 (56%), *p* < 0.001) (Table 1).

**Figure 2:**
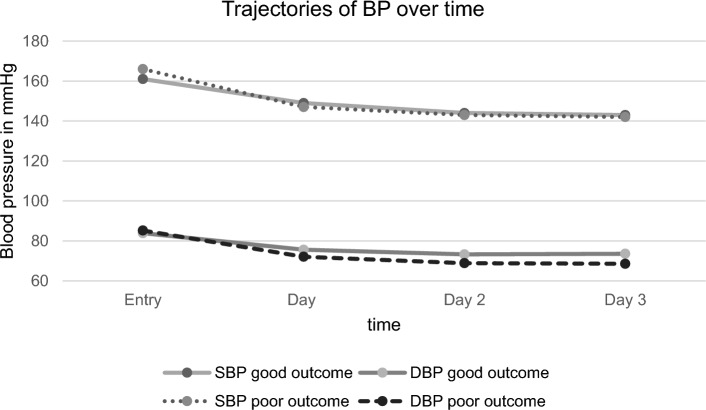
Trajectories of blood pressure over time of stroke patients regarding outcome at discharge. BP, blood pressure, SBP, systolic blood pressure; DBP, diastolic blood pressure.

SBP and DBP detected on admission (SBP_ad_; DBP_ad_) did not differ significantly between the group with good and poor functional outcome (mean SBP_ad_ 161 ± 28 mmHg vs 166 ± 36 mmHg; *p* = 0.23; mean DBP_ad_ 84 ± 17 mmHg vs 85 ± 22 mmHg; p = 0.58)), the same was true for the mean SBP measured during the first 72 h (SBP_72h_) at the stroke unit (mean SBP_72h_ 145 ± 15 mmHg vs 144 ± 18 mmHg; *p* = 0.55). Interestingly, mean DBP of the first 72 h (DBS_72h_) was significantly higher in patients with good outcome compared to those with poor outcome (mean DBP_72h_ 74 ± 10 mmHg vs 70 ± 13 mmHg; *p* < 0.001) (Table 3). A trajectory of SBP and DBP of both groups during the first 72 h after admission is outlined in Fig. 2. Mean SBP decrease during the first 72 h after admission did not differ significantly between patients with good and poor functional outcome (−16 ± 22 mmHg vs −22 ± 28 mmHg; *p* = 0.07). In contrast, mean DBP decrease over the first 72h after admission was significantly higher in patients with poor outcome compared to those with good outcome at discharge (−10 ± 15 mmHg vs −15 ± 21 mmHg; *p* = 0.012) (Table 3).

**Table 3:** Blood pressure characteristics of stroke patients regarding outcome at discharge. mRS, modified Rankin Scale, SBP, systolic blood pressure; DBP, diastolic blood pressure.

	All patients	Good outcome (mRS 0–2)	Poor outcome (mRS 3–6)	*p*-value
At entry (n = 422)				
SBP mmHg ± SD	163 ± 30	161 ± 28	166 ± 36	0.229^1^
DBP mmHg ± SD	84 ± 18	84 ± 17	85 ± 22	0.576^1^
SBP > 140 mmHg n (%)	341 (80.8)	255 (82.0)	86 (77.5)	0.370^2^
DBP > 90 mmHg n (%)	180 (42.7)	126 (40.5)	54 (48.6)	0.169^2^
Day 1 (n = 420)				
SBP mmHg ± SD	149 ± 20	149 ± 19	147 ± 22	0.353^1^
DBP mmHg ± SD	75 ± 13	76 ± 12	72 ± 14	0.023^1^
SBP > 140 mmHg n (%)	294 (70)	221 (71.3)	73 (66.4)	0.397^2^
DBP > 90 mmHg n (%)	64 (15.2)	45 (14.5)	19 (17.3)	0.591^2^
Day 2 (n = 418)				
SBP mmHg ± SD	143 ± 17	144 ± 16	143 ± 20	0.742^1^
DBP mmHg ± SD	72 ± 12	73 ± 12	69 ± 14	0.002^1^
SBP > 140 mmHg n (%)	260 (62.2)	192 (62.3)	68 (61.8)	0.999^2^
DBP > 90 mmHg n (%)	36 (8,6)	27 (8.8)	9 (8.2)	0.999^2^
Day 3 (n = 414)				
SBP mmHg ± SD	143 ± 17	143 ± 16	142 ± 19	0.872^1^
DBP mmHg ± SD	72 ± 12	74 ± 11	69 ± 14	0.001^1^
SBP > 140 mmHg n (%)	238 (57.5)	175 (57.6)	63 (57.3)	0.999^2^
DBP > 90 mmHg n (%)	30 (7.3)	22 (7.2)	8 (7.3)	0.999^2^
Ø 72 h (n = 420)				
SBP mmHg ± SD	145 ± 16	145 ± 15	144 ± 18	0.548^1^
DBP mmHg ± SD	73 ± 11	74 ± 10	70 ± 13	0.001^1^
BP decrease over 72 h, reference BP at entry				
SBP mmHg ± SD	−17.6 ± 24	−16.1 ± 22	−21.6 ± 28	0.069^1^
DBP mmHg ± SD	−11.1 ± 17	−9.7 ± 15	−15.3 ± 21	0.012^1^

^1^Calculated with t-Test. ^2^Calculated with Chi^2^-Test or Fishers-Exact-Test when expected frequencies were < 5.

**Figure 3:**
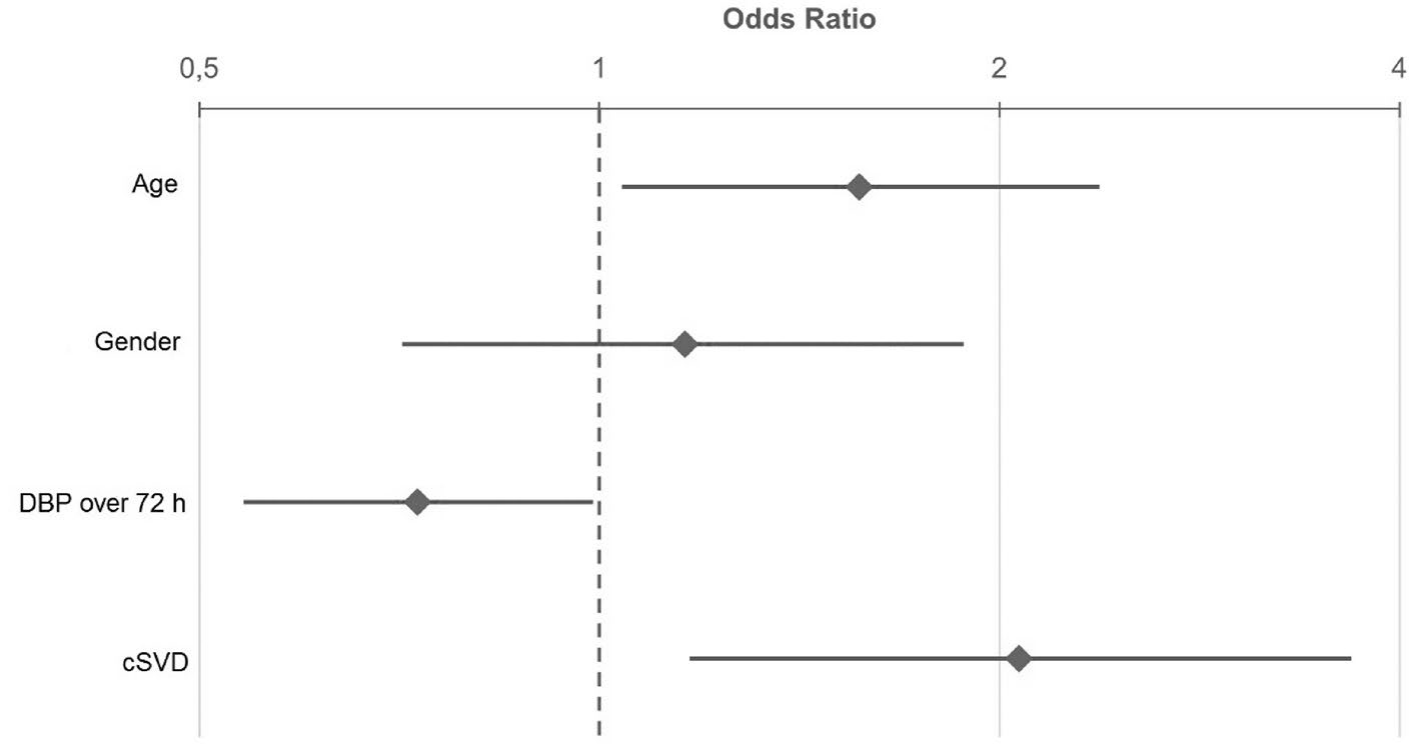
Odds Ratio and 95% confidence interval for age, gender, DBP over the first 72h after admission and cSVD of stroke patients regarding functional outcome at discharge. Odds Ratio for age and DBP over 72 h indicates the ratio of the odds from the third to the first quartile. Age: 1. Quartile = 61.5 years; 3. Quartile = 80 years; DBP over 72 h: 1. Quartile = 66 mmHg; 3. Quartile = 80.3 mmHg. DBP, diastolic blood pressure, cSVD, cerebral small vessel disease.

**Figure 4:**
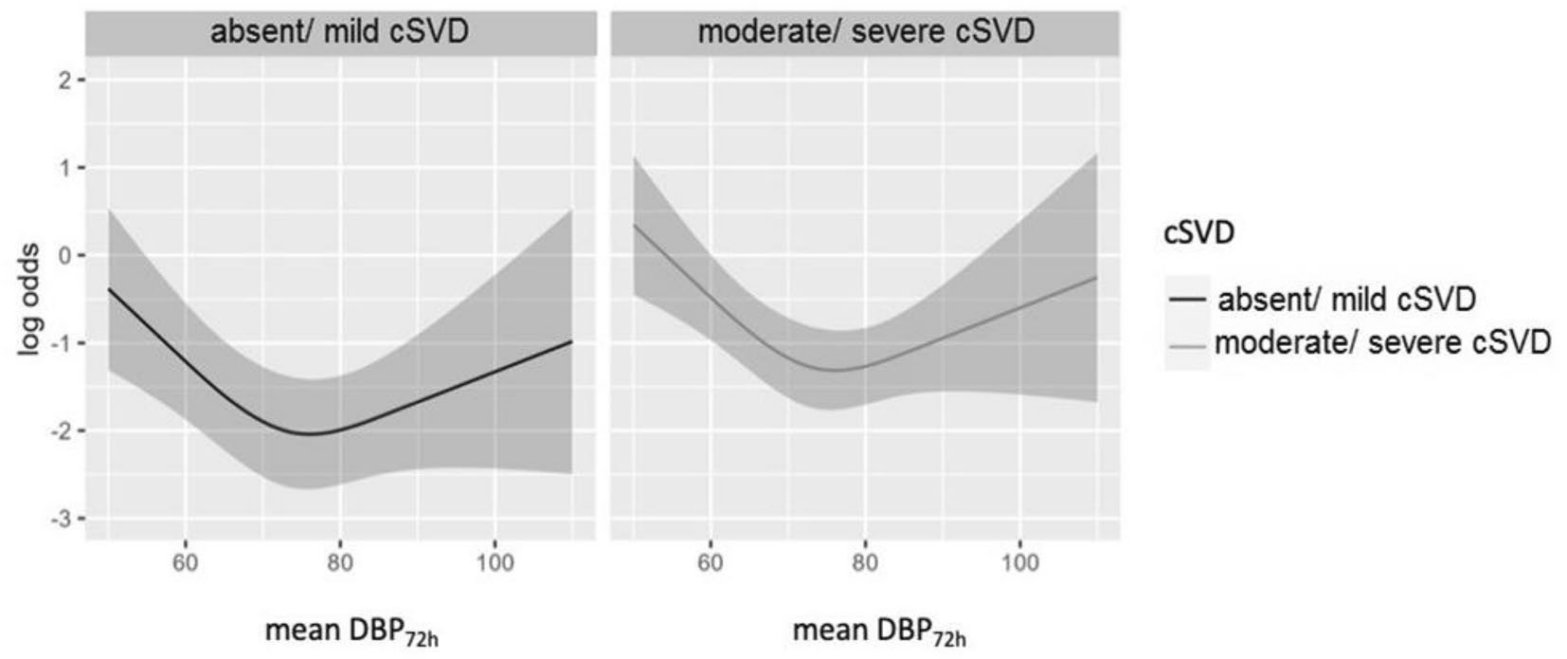
Log odds for cSVD dependent on DBP over the first 72 h after admission for patients regarding functional outcome at discharge. Adjusted for age and gender.

In multivariable logistic regression, significant predictors for poor clinical outcome at discharge were mean DBP measured during the first 72 h after admission (OR 0.73; 95%CI 0.54–0.99; *p* < 0.001) and cSVD (OR 2.07; 95%CI 1.17–3.68; *p* = 0.013), whereas the variable age and sex were not significantly associated with poor clinical outcome (Fig. 3). Additionally, we found a significant U-shaped dependence of the mean DBP_72h_ and clinical outcome (Fig. 4). Mean DBP < 66 mmHg and mean DBP > 80 mmHg over the first 72 h after admission were associated with a poor outcome at discharge.

One year after cerebrovascular event, new ischemic stroke or TIA was reported in 11.9% of patients with a good and in 9.89% of those with a poor clinical outcome at discharge (*p* = 0.62). Among patients with good and poor outcome, 5.78% and 12.9%, respectively, died within one year (*p* = 0.035).

### Late functional outcomes at 12-month follow-up

Twelve months after ischemic stroke or TIA, good functional outcome was found in 261 (70.7%) patients and poor outcome in 108 (29.3%) patients (Table 2). Among patients with a poor outcome, WMH (Fazekas score 2–3) were more frequent observed than in those with a good outcome at 12 months (74 (69.8%) vs 126 (48.8%), *p* < 0.001). The same was true for the presence of lacunes (56 (53.8%) vs 97 (37.7%), *p* = 0.007). CMB occurred in both, patients with poor and good outcome almost in the same frequency (35 (32.7%) vs 65 (26.1%), *p* = 0.24). Over all, the burden of cSVD was higher in patients with poor outcome compared to the group with good outcome at 12 months (84 (78.5%) vs 145 (55.6%), *p* < 0.001) (Table 2).

We found no significant difference between patients with good and poor functional outcome in respect to mean SBP_ad_ (161 ± 28 mmHg vs 166 ± 33 mmHg, *p* = 0.13) and mean DBP_ad_ (84 ± 16 mmHg vs 85 ± 22 mmHg, *p* = 0.73). SBP_72h_ was similar between patients with good and poor outcome at 12 months (mean SBP_72h_ 145 ± 16 mmHg vs 146 ± 16 mmHg; *p* = 0.86). In contrast, patients with a good outcome showed a significantly higher mean DBP_72h_ when comparing to patients with a poor outcome (mean DBP_72h_ 75 ± 10 mmHg vs 69 ± 10 mmHg; *p* < 0.001) (Table 4).

**Table 4:** Blood pressure characteristics of stroke patients regarding outcome at 12 months. mRS, modified Rankin Scale, SBP, systolic blood pressure; DBP, diastolic blood pressure.

	All patients	Good outcome (mRS 0–2)	Poor outcome (mRS 3–6)	*p*-value
At entry (n = 422)				
SBP mmHg ± SD	162 ± 30	161 ± 28	166 ± 33	0.133^1^
DBP mmHg ± SD	84 ± 18	84 ± 16	85 ± 22	0.725^1^
SBP > 140 mmHg n (%)	300 (81.3)	211 (80.8)	89 (82.4)	0.838^2^
DBP > 90 mmHg n (%)	157 (42.5)	111 (42.5)	46 (42.6)	0.999^2^
Day 1 (n = 420)				
SBP mmHg ± SD	149 ± 20	149 ± 19,4	148 ± 20	0.816^1^
DBP mmHg ± SD	75 ± 12	76 ± 12	71 ± 12	< 0.001^1^
SBP > 140 mmHg n (%)	260 (70.7)	184 (70.8)	76 (70.4)	0.999^2^
DBP > 90 mmHg n (%)	56 (15.2)	45 (17.3)	11 (10.2)	0.116^2^
Day 2 (n = 418)				
SBP mmHg ± SD	144 ± 17	144 ± 17	144 ± 17	0.621^1^
DBP mmHg ± SD	72 ± 12	74 ± 12	70 ± 11	< 0.001^1^
SBP > 140 mmHg n (%)	260 (62.2)	162 (62.5)	69 (63.9)	0.902^2^
DBP > 90 mmHg n (%)	36 (8.6)	25 (9.7)	5 (4.6)	0.164^2^
Day 3 (n = 414)				
SBP mmHg ± SD	143 ± 17	142 ± 16	144 ± 16	0.309^1^
DBP mmHg ± SD	72 ± 12	74 ± 11	68 ± 12	< 0.001^1^
SBP > 140 mmHg n (%)	238 (57.5)	143 (55.9)	68 (63.0)	0.255^2^
DBP > 90 mmHg n (%)	30 (7.3)	19 (7.4)	7 (6.5)	0.924^2^
Ø 72 h (n = 420)				
SBP mmHg ± SD	145 ± 16	145 ± 16	146 ± 16	0.683^1^
DBP mmHg ± SD	73 ± 11	75 ± 10	70 ± 10	< 0.001^1^
BP decrease over 72 h, reference BP at entry				
SBP mmHg ± SD	−17.3 ± 23	−15.8 ± 22	−20.6 ± 26	0.090^1^
DBP mmHg ± SD	−11.1 ± 17	−9.4 ± 15	−15.5 ± 21	0.007^1^

^1^Calculated with t-Test. ^2^Calculated with Chi^y^-Test or Fishers-Exact-Test when expected frequencies were < 5.

Mean SBP decrease during the first 72 h after admission did not show a statistic difference between patients with good and poor functional outcome (−16 ± 22 mmHg vs −21 ± 26 mmHg; *p* = 0.09). However, mean DBP decrease over the first 72 h after admission was significantly higher in patients with poor outcome compared to those with good outcome at 12 months (−9 ± 15 mmHg vs −15 ± 21 mmHg; *p* = 0.007) (Table 4).

**Figure 5:**
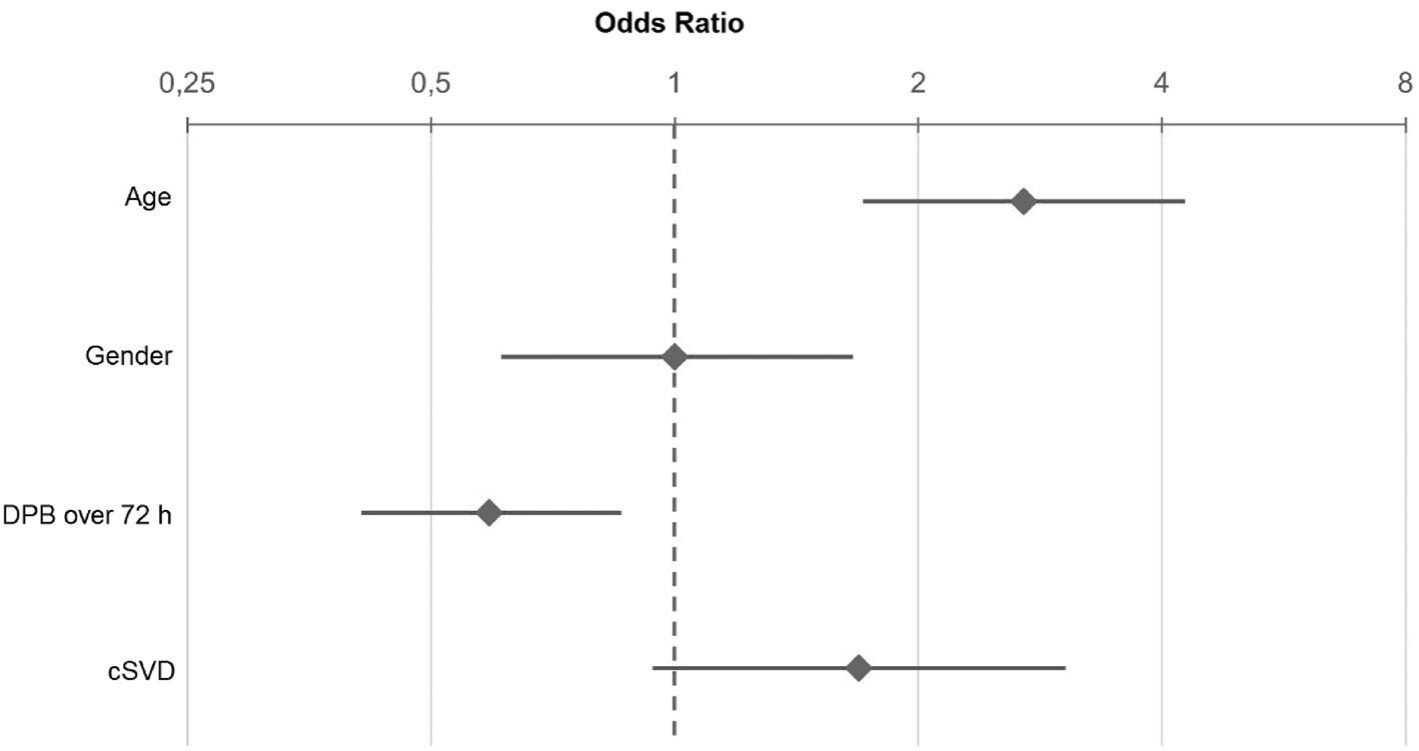
Odds Ratio and 95% confident interval for age, gender, DBP over the first 72 h after admission and cSVD of stroke patients regarding outcome at 12 months. OR for age and DBP over 72 h indicates the ratio of the odds from the third to the first quartile. Age: 1. Quartile = 61.5 years; 3. Quartile = 80 years; DBP over 72 h: 1. Quartile = 66 mmHg; 3. Quartile = 80.3 mmHg. DBP, diastolic blood pressure, cSVD, cerebral small vessel disease.

In multivariable logistic regression, significant predictors for poor clinical outcome at 12 months are mean DBP_72h_ (OR 0.59; 95%CI 0.41–0.86; *p* = 0.018) and age (OR 2.7; 95%CI 1.71–4.27; *p* < 0.001) (Fig. 5). The burden of cSVD did not significantly predict poor outcome at 12 months; nonetheless, there was a statistical trend (OR 1.69; 95%CI 0.94–3.04; *p* = 0.08) (Fig. 5).

Twelve months after cerebrovascular event, recurrent ischemic stroke or TIA was diagnosed in 9.58% of patients with a good outcome and in 15.9% of those with a poor clinical outcome (*p* = 0.09).

## Discussion

This prospective study of 423 patients with an AIS or TIA showed that cSVD burden was significantly higher in patients with a poor functional outcome at discharge and 12 months after cerebrovascular event. However, neither SBP_ad_ nor mean SBP_72h_ was significantly related to functional outcome at discharge or at 12 months after index event. An unexpected finding of the present study was a significant association between mean DBP_72h_ and functional outcome at discharge and 12 months afterwards.

A growing number of studies suggests that cSVD in patients with AIS is independently associated with poor functional outcome in the short and long term after cerebrovascular event^32–34^ which is in line with our findings. The impact of cSVD on functional outcome after AIS is poorly understood. It has been shown that severe cSVD may lead to a reduction of cerebral blood flow and is associated with impaired cerebral autoregulation^23,35,36^. Thus, one might argue that low SBP during the acute phase of IS may result in a drop in cerebral blood flow of those patients with severe cSVD. A low SBP might lead to an even faster and more extensive damage of cerebral tissue surrounding the infarcted core than a high SBP, which finally may result in a poor functional outcome. Though, we did not find a statistically significant relationship between SBP_ad_ or SBP_72h_ and functional outcome in stroke patients with or without cSVD. This might be due to the small sample size in this study. Additionally, we included patients with AIS as well as with TIA who were treated for hypertension; furthermore, some patients had a mRS > 0 prior to study enrollment, which might—to some extent—influence the results. Another reason for the missing effect of SBP on functional outcome in patients with cSVD could be the fact that SBP only impacts functional outcome when cerebral perfusion is severely compromised as one would expect in patients with high burden of cSVD (e.g., Fazekas grade 3, multiple lacunes and/or CMB). Importantly, we combined patients with Fazekas grade 2 and 3 in one group which could have biased the results. In addition, cSVD burden was determined by using a qualitative and not by a quantitative approach, i.e., we did not assess the volume of WMH in our patients. However, a higher Fazekas scale score is highly correlated with higher WMH volume^37^. Furthermore, different regional distribution of WMH load and/or locations of stroke lesions might also impact functional outcome^38^. Furthermore, factors independent of blood pressure such as variations in the cerebral autoregulation, especially in the acute phase as well as genetics are also of great importance in this context. On the other hand, a large randomized trial (n = 3035) has also revealed that SBP is not likely to explain a relationship between cSVD and poor functional outcome after stroke when adjusting for age^39^. In contrast to our study, the main focus of the aforementioned study was on SBP variability during the first 24 h after stroke.

Although SBP decreased over the first 72 h after admission, there was no statistically significant relationship between SBP decline and functional outcome at discharge and at 12 months after AIS, and thus, an interaction between these parameters and cSVD burden was not expected. Our results are in contrast with findings of a previously published study, showing that a large change of SBP over the first 24 h after stroke is related with a poor functional outcome in the short term (i.e. at day 7 after stroke) as well as in the long term (90 days after stroke)^21^. However, patients with cSVD burden were not particularly addressed in that study. Although all patients have been recommended to take continuously antihypertensive drugs, adherence of drug intake is low; nearly a quarter of patients are non-adherent to their cardiovascular medicaments^40–42^. Hence, repeated high SBP values (> 150 mmHg) during the 12 months might occur in these stroke patients and further impair cerebral tissue which has been already damaged by pre-existing cSVD. This might further bias our results.

Little is known about the impact of DBP on functional outcome in stroke survivors with and without cSVD. Here, we found that mean DBP_72h_ was significantly lower in stroke patients with cSVD. Moreover, DBP_72h_ and cSVD burden were associated with a poor outcome at discharge. In contrast to our findings, a previously published trial revealed no consistent association between the level of DBP measured over 24 h and functional outcome^21^, however, in the aforementioned trial, DBP was generally lower in patients with a poor outcome at day 7 and at day 90^21^. In another previously published study, DBP variability was associated with poor functional outcome at hospital discharge^43^. However, the aforementioned studies did not focus on stroke patients with/without cSVD. Göthel-Ezzeiani and coworkers also investigated the impact of WMH and blood pressure on functional outcome. They found a relationship between DBP and mortality but none between DBP and functional outcome at 3 months^44^; of note, only stroke patients undergoing mechanical recanalization were included in their study. In the present study, there was a significant linear relationship between DBP_72h_ and late functional outcome (at 12 months) but only a statistical trend for cSVD burden when adjusting for sex and age. In contrast, a previous trial reported similar DBP measured over 24 h after stroke in patients with good and poor late functional outcome (at 3 months)^45^. However, that study did not specifically investigate stroke survivors presenting cSVD and enrolled only stroke patients treated with intravenous thrombolysis.^22^,^22^

Additionally, we observed a U-shaped relationship between mean DBP_72h_ and functional outcome at discharge among stroke patients with and without cSVD meaning that only a DBP of about 60–80 mmHg was related to a better functional outcome. A U-shaped relationship between DBP on admission and early deterioration as well as functional outcome at 3 months^4^ or 6 months^46^ was provided by previous studies. In contrast to our study, the cutoff value for DBP was higher, namely at 100–110 mmHg^4^ or 87.8–95 mmHg, respectively^46^. In the latter study however, this association was no longer significant, when assessed with logistic regression. Other large studies on patients with ischemic stroke (n > 300′000 patients)^47^ or TIA (n > 200′000 patients)^48^ reported a U-shaped relationship between DBP on admission and independent ambulation at discharge, likelihood of being discharged at home, and in-hospital death such that below and above 70 mmHg the unadjusted and adjusted odds of these outcomes increased. This value is in the same range as observed in our study. In a cohort study of patients with ischemic stroke treated with endovascular therapy, DBP on admission and functional outcome at 3 months showed a J-shaped relationship with an inflection point at the median value of DBP of 81 mmHg^49^. In contrast to our work, cSVD was not in the scope of these studies.

The reason why there was a U-shaped relationship between DBP and functional outcome whereas this was not true for SBP in the present study remains elusive. One possible explanation of this observation could be the small number of patients. The U-shaped association between low and high DBP and poor functional outcome might be closely linked to the state of cerebral perfusion for several reasons. First of all, there is some evidence that cerebral perfusion might be lower in patients with arterial hypertension irrespective of the presence of WMH compared to healthy controls^50^. Patients with WMH have been reported to exhibit a reduction of cerebral blood flow of the white matter (up to 38%) compared with those without WMH^51,52^. There was even a decrease in CBF within normal appearing white matter surrounding the WMH^53^. On the other hand, levels of cerebral perfusion especially in the early phase of AIS display a reverse U-shaped curve depending on SBP and DBP levels and—in turn—impact functional outcome at 3 months after stroke^54^. The most favorable functional outcome was associated with a SBP between 161 to 177 mmHg and DBP ranging from 103 to 114 mmHg^54^. Interestingly, Park and Ovbiagele found also an independent association between DBP and vascular outcomes 2 years after non-cardioembolic stroke, showing that DBP < 70 mmHg as well as DBP > 90 mmHg was linked to an increased risk of vascular events^55^. Although this study revealed that patients with low DBP had higher comorbidities of diabetes mellitus, heart failure and carotid artery disease, DBP < 70 mmHg (but not DBP > 90 mmHg) was an independent predictor of vascular events, in particular recurrent stroke, after multivariable adjustment^55^. Notably, when the BP in the brachial artery is 117/75 mmHg in normotensive subjects, the pressure in the lenticulostriate vascular bed would be 91/58 mmHg and that in the same-size parietal arterioles 59/38 mmHg according to the calculation of Blanco and coworkers^56^. Given that there is a BP gradient in the brain, a decrease in peripheral DBP may result in a critical undersupply of cerebral tissue, since more than half of cerebral perfusion is during diastole^57^.

The present study has some limitations. First, the sample size of this work is relatively small. Second, we included patients receiving intravenous thrombolysis and/or undergoing mechanical thrombectomy as well as those with neither of these acute stroke therapies. However, this reflects the real, less than ideal clinical everyday practice. Third, the patients showed mild symptoms of stroke making a generalization of the study data difficult. Fourth, no additional cerebral MRI was performed at 12 months (except in patients with recurrent stroke or TIA) and thus, new or enlarged WMH were not able to be detected. Notably, WMH may regress over time after stroke and thus, provide potentially better functional and brain tissue outcome^58^. The strength of this work are the prospective study design and well-characterized patients. Furthermore, we used mean SBP and DBP over the first 72 h along with BP on admission. In contrast, a plenty of studies investigating the effect of acute-phase BP on functional outcome after AIS have used a single BP value (mainly the BP on admission) or BP values over the first 24 h as a predictor for the functional outcome. However, different mechanisms may be responsible for an elevation of BP values measured on admission. Beside a physiological response to cerebral ischemia, untreated arterial hypertension as well as increased sympathetic activation, fear of serious illness and hospitalization may contribute to increased BP values on admission^59,60^. Moreover, data collection and functional assessment by means of a structured telephone interview were carried out by one mRS-experienced person (S. G.).

In conclusion, this observational prospective study demonstrates that cSVD predicts a poor outcome among stroke survivors and thus corroborates findings of previous studies. We did not find a statistically significant relationship between cSVD, level of SBP, and functional outcome probably due to the fact that SBP_ad_ and SBP_72h_ values ranged between 145 and 160 mmHg, values that have been associated with a good functional outcome after AIS. However, DBP and cSVD were associated with poor outcome depending on the level of DBP; additionally, DBP showed a U-shaped relationship with functional outcome. When comparing our work to other studies which investigate the impact of BP on stroke outcome, a lot of them only focus on the effect of SBP and reperfusion grade after mechanical thrombectomy, without including cSVD^21,43–45^, or refer to cSVD alone^32–34^, making comparisons with our study difficult. Here, we have examined the impact of BP in relating with cSVD on stroke outcome in the acute phase, that has been rarely investigated from this point of view. Furthermore, our study suggests a prognostic significance for functional outcome after stroke depending on DBP value and degree of cSVD burden, which has scarcely been addressed so far, since most studies on stroke outcome analyze only the significance of SBP. Our findings may suggest an individualized stroke care by either lowering or elevating DBP in order to influence functional outcome. However additional large randomized studies are required to further investigate an association between DBP, cSVD and functional outcome after IS.

## Data Availability

All data generated or analyzed during this study are included in this published article.
